# Assessment of corneal biomechanics, tonometry and pachymetry with the Corvis ST in myopia

**DOI:** 10.1038/s41598-020-80915-9

**Published:** 2021-02-04

**Authors:** Xiaorui Wang, Colm McAlinden, Hongbing Zhang, Jie Yan, Dan Wang, Wei Wei, Shengjian Mi

**Affiliations:** 1 Department of Ophthalmology, The First Affiliated Hospital of Xi’an Jiaotong University, No. 277, West Yanta Road, Xi’an, Shaanxi China; 2 Laser Vision Center, The First Affiliated Hospital of Northwestern University, No. 30, Fenxiang Alley, Xi’an, Shaanxi China; 3 Shaanxi Institute of Ophthalmology, Xi’an, China; 4 Department of Ophthalmology, Singleton Hospital, Swansea Bay University Health Board, Swansea, UK

**Keywords:** Biomarkers, Medical research

## Abstract

To evaluate the repeatability of Corvis ST corneal biomechanical, tonometry and pachymetry measurements, and agreement of pachymetry measures with the Pentacam HR and RTVue OCT. Three consecutive measurements of the right eye of 238 myopic subjects were acquired with the Corvis ST, Pentacam HR, and RTVue OCT. Repeatability of Corvis ST was evaluated by within-subject standard deviation [S_w_] and repeatability limit [*r*]. The agreement of central corneal thickness (CCT) measurements were compared among the three instruments using the Bland–Altman limits of agreement. Comparisons were further stratified by CCT (Cornea_thin_ ≤ 500 µm; Cornea_normal_ = 500–550 µm; Cornea_thick_ > 550 µm). S_w_ was below 1 mmHg in Cornea_thin_, Cornea_normal_, and Cornea_thick_ groups for IOP and bIOP. S_w_ for SP-A1 were 4.880, 6.128, 7.719 mmHg/mm respectively. S_w_ for CBI were 0.228, 0.157, 0.076, and correspondingly S_w_ for TBI and SSI were 0.094 and 0.056, 0.079 and 0.053, 0.070 and 0.053. The Bland–Altman plots for CCT implied poor agreement with mean differences of 29.49 µm between Corvis and OCT, 9.33 µm between Pentacam and OCT, and 20.16 µm between Corvis and Pentacam. The Corvis ST showed good repeatability with the exception of CBI in the various CCT groups. The CCT measured by Corvis ST was not interchangeable with Pentacam HR and RTVue OCT.

## Introduction

Corneal biomechanics play an important role in the diagnosis and characterization of ocular diseases such as corneal ectasia, evaluation for corneal laser refractive surgery candidacy, and post refractive surgery monitoring^1–3^. It is gradually recognized that the corneal topographic changes in keratoconus are most likely secondary to a focal weakening that initiates a biomechanical decompensation^4^. Corneal biomechanics is also important in glaucoma management, particularly in normal tension glaucoma^5, 6^.

Biomechanical properties of the cornea can be evaluated as the response of the cornea under certain stress. The measurement of a linearly elastic material can be determined by a single elastic modulus, defined by the slope of the stress–strain plot, which describes how much stress will deform the material under specific conditions. However, the cornea is a viscoelastic tissue, which is not a typical type of linear elastic material. Its biomechanical complexity is enhanced by several important biomechanical concepts^7^. In the cornea, the measured modulus is determined by not only the magnitude of the applied force, but also on the rate at which it is applied; a faster strain rate produces a stiffer corneal response^8^. Moreover, the stress–strain relationship of the cornea can also be affected by intraocular pressure (IOP) and corneal thickness^9–11^. There are some technologies now available for the measurement of the ocular biomechanical response, including air-jet infrared light technology (Ocular Response Analyzer, ORA; Reichert, Inc., Depew, NY, USA), air-puff Scheimpflug imaging system (Corvis Scheimplug Tonometer, Corvis ST, Oculus Optikgeräte GmbH, Wetzlar, Germany), air-puff optical coherence tomographer (OCT) (not commercially available) and air-puff biometry (not commercially available)^12, 13^.

The dynamic Scheimpflug imaging analysis system, Corvis ST (Oculus Optikgeräte GmbH, Wetzlar, Germany), is a device allowing visualization and measurement of the corneal biomechanical response to a standardized air puff pressure concurrently. Following the air puff, the cornea moves inwards until it reaches maximum deformation and then rebounds back to its original shape. Using the Corvis ST, the deformation of the central 8 mm of the cornea along the horizontal meridian is recorded using a high-speed Scheimpflug camera at a rate of 4330 frames per second. Within 31 ms, the Corvis ST acquires 140 images of the cornea with a high resolution of 640 × 480 pixels. High-speed imaging allows for analysis of the detailed movement of the cornea during the deformation process, thus may provide clinically relevant parameters correlated with the biomechanical properties of the cornea.

As research using the Corvis ST advances, new parameters have been developed. The latest software version (V.1.6r2015) offers a total of 25 parameters, including dynamic corneal response (DCRs), Vinciguerra screening data and biomechanical/tomographic measurement and an promising parameter of Stress–Strain Index (SSI) which seems to be independent of IOP and corneal thickness. An ideal device for measuring corneal biomechanics should provide precise (repeatable and reproducible) measurements. The current study aimed to evaluate the repeatability of measurements acquired with the Corvis ST and investigate whether repeatability varied across different CCT thicknesses. Finally, the agreement of CCT measurements using the Corvis ST with the Pentacam HR (Oculus Optikgeräte GmbH, Wetzlar, Germany, software version.1.2r43) and the RTVue OCT (Optovue Inc., Fremont, California, USA, software version.2018.0.0.18) centered on the corneal apex were compared.

## Patients and methods

This was a prospective observational study. Patients presenting for refractive surgery (no previous corneal laser surgery history) at the First Affiliated Hospital of Xi’an Jiaotong University between April and August 2019 were invited to participate in the current prospective study. Exclusion criteria included the following conditions: rigid lens wear, currently pregnant or nursing, and a history of ocular surgery. Soft lens wearers were required to cease contact lens wear for at least 7 days prior to measurements, IOP > 21 mmHg or IOP < 10 mmHg, keratoconus and other corneal pathologies. The study was approved by the Ethics Board of the First Affiliated Hospital of Xi’an Jiaotong University Institute, and was performed in compliance with the tenets of the Declaration of Helsinki. All participants signed an informed consent form before participation in the study.

All subjects underwent comprehensive ophthalmic examinations, including slit-lamp biomicroscopy, fundoscopy, auto-refraction (AR-1, NIDEK, Japan), subjective manifest refraction with an undilated pupil (RT-500, NIDEK, Japan), and Scheimpflug corneal topography with the Pentacam HR (Oculus Optikgeräte GmbH, Wetzlar, Germany, software version.1.2r43), and anterior segment OCT (RTVue, Optovue Inc., Fremont, California, USA, software version.2018.0.0.18). All measurements were acquired with pachymetry mode centered on the corneal vertex normal (apex). Three consecutive measurements were performed on each eye using the Corvis ST (Oculus Optikgeräte GmbH, Wetzlar, Germany, software version. V.1.6r2015) by the same experienced technician. Only image quality graded as “OK” were collected. All the examinations were performed between 10:00 and 17:00. For each patients, all examinations were conducted within 1 h.

Two hundred and thirty eight patients (112 females and 126 males, mean age 25.41 ± 7.44 years, range 18 to 51) were enrolled. Only the right eye of each patient was included in the analyses to avoid the bias of the bilateral eye correlation. To evaluate the possible influence of CCT on the performance of the Corvis ST, subjects were divided into three groups based on the CCT measured with the anterior segment OCT: thin cornea (Cornea_thin_, CCT ≤ 500 µm), normal thickness cornea (Cornea_normal_, CCT = 500-550 µm), and thick cornea (Cornea_thick_, CCT > 550 µm).

Statistical analyses were performed using SPSS version 24.0 (IBM, Armonk, New York, USA). Descriptive data were expressed as mean ± standard deviation (SD). The Shapiro–Wilk test was used to assess if data were normally distributed. To determine the repeatability of the Corvis ST measurements, the within-subject standard deviation (S_w_) of three consecutive measurements was calculated. The repeatability limit was also calculated and is defined as 1.96√2 × S_w_^14, 15^. Bland–Altman plots were used to assess the measurement agreement of CCT among the three devices. For the comparison of biomechanical characters among the three corneal thickness groups, a linear mixed model analysis of covariance (ANCOVA) was used to adjust for the effect of age and spherical equivalent (SE).

## Results

### Patient demographics

**Figure 1:**
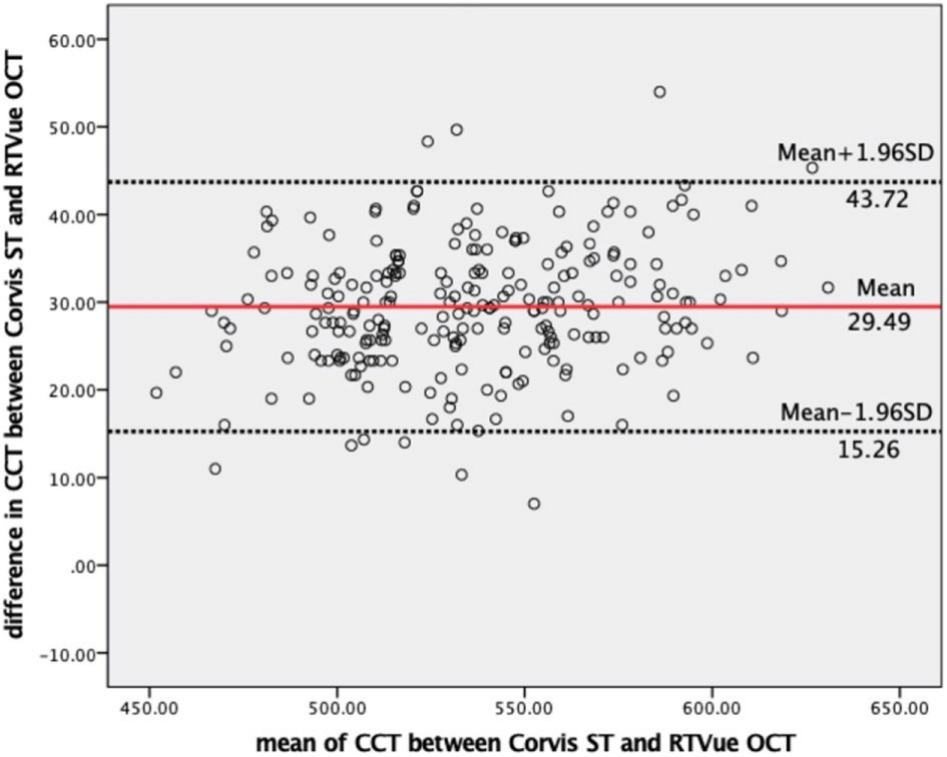
Bland–Altman plots show the agreement of CCT between the Corvis ST and RTVue OCT. The solid red line represents the mean difference, and the black dotted lines represent the 95% limits of agreement.

**Figure 2:**
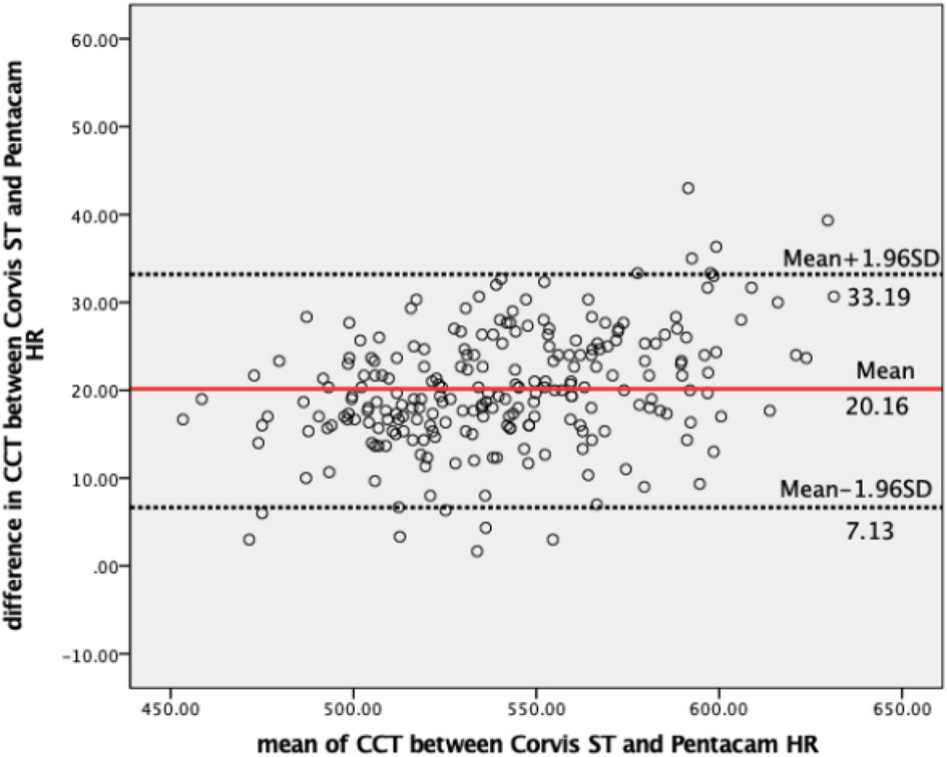
Bland–Altman plots show the agreement of CCT between the Corvis ST and Pentacam HR. The solid red line represents the mean difference, and the black dotted lines represent the 95% limits of agreement.

**Figure 3:**
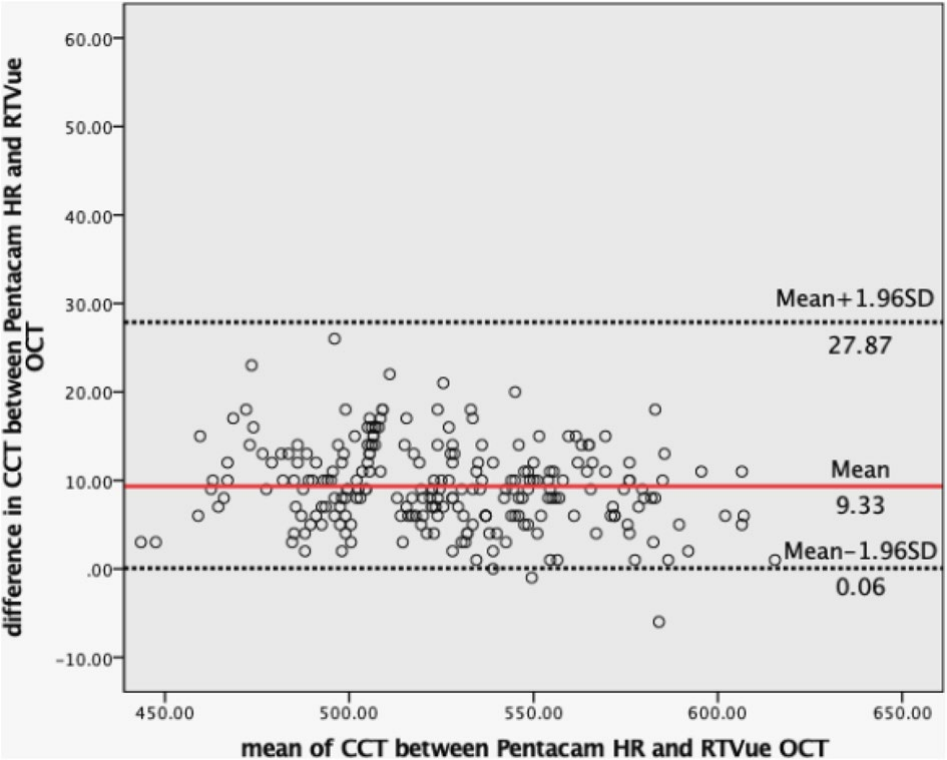
Bland–Altman plots show the agreement of CCT between the Pentacam HR and RTVue OCT. The solid red line represents the mean difference, and the black dotted lines represent the 95% limits of agreement.

The mean age, SE, axial length (AL), mean K value (Km), corneal diameter, IOP, bIOP, central corneal thickness by OCT [CCT(o)], central corneal thickness by Corvis ST [CCT(c)], and central corneal thickness by Pentacam HR [CCT(p)] for the study subjects are listed in Table 1. Subjects in the Cornea_thin_ group were significantly older than the other two groups. The eyes in this group were also more myopic and had slightly longer axial lengths (AL). CCT with both OCT and Corvis ST were significant different among the three groups (P < 0.001) (Figs. 1, 2, 3). No significant differences in Km or corneal diameter were detected among those three groups.

**Table 1:** Characteristics of the study subjects.

	Cornea_thin_ (n = 91)	Cornea_normal_ (n = 94)	Cornea_thick_ (n = 53)	P
Age, years	27.49 ± 8.14	24.04 ± 6.41	24.26 ± 7.21	< 0.05*
SE, D	− 5.98 ± 2.09	− 5.25 ± 2.45	− 5.29 ± 2.21	< 0.05*
Axial length, mm	25.89 ± 1.15	25.63 ± 1.09	25.41 ± 2.25	> 0.05
Km, D	43.41 ± 1.28	43.26 ± 1.35	43.19 ± 1.28	> 0.05
Corneal diameter, mm	11.52 ± 0.41	11.44 ± 0.39	11.42 ± 0.37	> 0.05
IOP, mmHg	14.00 ± 1.57	14.93 ± 1.75	16.31 ± 1.79	< 0.001*
CCT(o), µm	486.14 ± 14.72	528.33 ± 11.68	570.25 ± 16.01	< 0.001*
CCT(c), µm	515.30 ± 16.89	557.16 ± 13.35	601.48 ± 17.89	< 0.001*
CCT(p), µm	497.00 ± 15.84	537.01 ± 11.71	578.74 ± 16.48	< 0.001*

*SE* spherical equivalent, *Km* mean K value, *CCT(o)* central corneal thickness by anterior OCT, *CCT(c)* central corneal thickness by Corvis ST, *CCT(p)* central corneal thickness by Pentacam HR.

One-way ANOVA, post-hoc test of LSD was used, for age: P_thin-normal_ = 0.001, P_thin-thick_ = 0.011, P_normal-thick_ = 0.086; SE: P_thin-normal_ = 0.031, P_thin-thick_ = 0.082, P_normal-thick_ = 0.916; axial length: P_thin-normal_ = 0.220, P_thin-thick_ = 0.056, P_normal-thick_ = 0.379; for axial length, Km and corneal diameter, All P_thin-normal_, P_thin-thick_, P_normal-thick_ > 0.05; and for CCT(o), CCT(c) and CCT(p), All P_thin-normal_, P_thin-thick_, P_normal-thick_ < 0.001.

*Statistical significant (P < 0.05).

### The repeatability of Corvis ST measurements

In all three corneal thickness groups, the majority of parameters including IOP, bIOP, CCT, DA ratio 2, SP-A1, Defle A, A1V, A1T, A2V, A2T, PD, Def A showed good repeatability (Table 2). In thick corneas, the repeatability of the Corvis ST measurement was slightly better than thin and normal corneas (Table 2).

**Table 2:** The measurement repeatability of Corvis ST in thin, normal and thick corneas.

	Mean ± SD	S_w_	Repeatability limit, *r*
**IOP, mmHg**			
Cornea_thin_	14.00 ± 1.57	0.975	2.701
Cornea_normal_	14.93 ± 1.75	0.997	2.761
Cornea_thick_	16.31 ± 1.79	0.897	2.485
**bIOP, mmHg**			
Cornea_thin_	14.72 ± 1.48	0.948	2.627
Cornea_normal_	14.60 ± 1.55	0.957	2.650
Cornea_thick_	14.72 ± 1.57	0.777	2.153
**CCT(c), µm**			
Cornea_thin_	515.30 ± 16.89	6.165	17.078
Cornea_normal_	557.16 ± 13.35	6.640	18.393
Cornea_thick_	601.48 ± 17.89	7.658	21.213
**DA ratio 2**			
Cornea_thin_	4.99 ± 0.42	0.205	0.568
Cornea_normal_	4.51 ± 0.33	0.232	0.644
Cornea_thick_	4.13 ± 0.29	0.139	0.386
**IR, mm**^**−1**^			
Cornea_thin_	9.27 ± 0.87	0.403	1.117
Cornea_normal_	8.57 ± 0.91	0.514	1.423
Cornea_thick_	7.55 ± 0.76	0.360	0.996
**ARTh**			
Cornea_thin_	431.92 ± 77.08	59.239	164.090
Cornea_normal_	456.55 ± 91.09	53.466	148.100
Cornea_thick_	527.10 ± 92.06	61.090	169.219
**SP-A1, mmHg/mm**			
Cornea_thin_	79.21 ± 9.04	4.880	13.519
Cornea_normal_	91.56 ± 9.79	6.128	16.974
Cornea_thick_	102.54 ± 9.27	7.719	21.380
**Arc length, mm**			
Cornea_thin_	− 0.13 ± 0.02	0.012	0.034
Cornea_normal_	− 0.14 ± 0.02	0.014	0.039
Cornea_thick_	− 0.15 ± 0.02	0.013	0.036
**Defle A, mm**			
Cornea_thin_	1.00 ± 0.09	0.035	0.098
Cornea_normal_	0.96 ± 0.09	0.036	0.099
Cornea_thick_	0.90 ± 0.08	0.032	0.089
**DefleA ratio 2**			
Cornea_thin_	6.55 ± 0.77	0.541	1.499
Cornea_normal_	5.73 ± 0.71	0.570	1.578
Cornea_thick_	5.18 ± 0.72	0.390	1.080
**EMo, mm**			
Cornea_thin_	0.27 ± 0.07	0.041	0.125
Cornea_normal_	0.26 ± 0.06	0.031	0.086
Cornea_thick_	0.26 ± 0.06	0.030	0.083
**ICR, mm**^**−1**^			
Cornea_thin_	0.16 ± 0.01	0.007	0.019
Cornea_normal_	0.16 ± 0.02	0.009	0.025
Cornea_thick_	0.14 ± 0.01	0.007	0.019
**A1L, mm**			
Cornea_thin_	2.19 ± 0.22	0.313	0.868
Cornea_normal_	2.27 ± 0.22	0.302	0.836
Cornea_thick_	2.45 ± 0.17	0.283	0.783
**A1V, ms**^**−1**^			
Cornea_thin_	0.16 ± 0.02	0.010	0.029
Cornea_normal_	0.15 ± 0.02	0.011	0.030
Cornea_thick_	0.14 ± 0.02	0.010	0.028
**A1T, ms**			
Cornea_thin_	7.34 ± 0.24	0.145	0.402
Cornea_normal_	7.53 ± 0.26	0.147	0.408
Cornea_thick_	7.68 ± 0.28	0.154	0.427
**A2L, mm**			
Cornea_thin_	1.76 ± 019	0.309	0.857
Cornea_normal_	1.89 ± 0.23	0.275	0.761
Cornea_thick_	2.19 ± 0.29	0.339	0.940
**A2V, ms**^**−1**^			
Cornea_thin_	− 0.29 ± 0.02	0.018	0.050
Cornea_normal_	− 0.29 ± 0.03	0.018	0.051
Cornea_thick_	− 0.28 ± 0.02	0.016	0.044
**A2T, ms**			
Cornea_thin_	22.11 ± 0.30	0.180	0.499
Cornea_normal_	22.11 ± 0.35	0.200	0.554
Cornea_thick_	22.00 ± 0.34	0.202	0.559
**HCT, ms**			
Cornea_thin_	17.28 ± 0.24	0.309	0.855
Cornea_normal_	17.31 ± 0.26	0.295	0.818
Cornea_thick_	17.30 ± 0.28	0.265	0.734
**PD, mm**			
Cornea_thin_	5.13 ± 0.21	0.110	0.306
Cornea_normal_	5.06 ± 0.24	0.098	0.272
Cornea_thick_	4.94 ± 0.23	0.101	0.280
**HCR, mm**^**−1**^			
Cornea_thin_	6.54 ± 0.54	0.361	1.000
Cornea_normal_	6.88 ± 0.62	0.570	1.579
Cornea_thick_	7.52 ± 0.54	0.548	1.518
**Def A, mm**			
Cornea_thin_	1.14 ± 0.10	0.043	0.119
Cornea_normal_	1.11 ± 0.10	0.050	0.113
Cornea_thick_	1.05 ± 0.08	0.033	0.091
**CBI**			
Cornea_thin_	0.55 ± 0.31	0.228	0.632
Cornea_normal_	0.27 ± 0.26	0.157	0.435
Cornea_thick_	0.05 ± 0.10	0.076	0.210
**TBI**			
Cornea_thin_	0.52 ± 0.24	0.094	0.261
Cornea_normal_	0.33 ± 0.26	0.079	0.218
Cornea_thick_	0.23 ± 0.21	0.070	0.195
**SSI**			
Cornea_thin_	0.87 ± 0.14	0.056	0.157
Cornea_normal_	0.88 ± 0.13	0.053	0.148
Cornea_thick_	0.96 ± 0.13	0.053	0.147

*IOP* intraocular pressure, *bIOP* biomechanically corrected IOP, *CCT(c)* central corneal thickness by Corvis ST, *DA ratio 2* deformation amplitude ratio(2 mm), *IR* Integrated radius (mm^−1^), *ARTh* Ambrósio’s relational thickness, *SP-A1* stiffness parameter at first applanation, *Arc length* the change of arc length during the deformation response within 3.5 mm horizontal distance from the apex both nasally and temporally, *Defle A* deflection amplitude, *DefleA ratio 2* deflection amplitude ratio at 2 mm, *EMo* whole eye movement, *ICR* inverse concave radius, *A1L* applanation 1 length, *A1V* applanation 1 velocity, *A1T* applanation 1 time, *A2L* applanation 2 length, *A2V* applanation 2 velocity, *A2T* applanation 2 time, *HCT* time to highest concavity, *PD* peak distance, *HCR* highest concavity radius, *Def A* deformation amplitude, *CBI* Corvis biomechanical index, *TBI* Tomographic and biomechanical index, *SSI* Stress strain index *SD* standard deviation, *Sw* within-subject standard deviation, *r* repeatability limit.

### Intergroup comparison of the Corvis ST measurement

After adjusting for the differences in age and SE, comparisons of Corvis ST measurements among the three groups showed that the majority of parameters varied according to the corneal thickness, except for bIOP, EMo, A2T and HCT. Thicker corneas were associated with lower DA ratio, IR, Defle A, Defle A ratio, ICR, A1V, A2V, PD, Def A, CBI, and TBI, as well as higher ARTh, SP-A1, Arc length, A1L, A1T, A2L, and HCR (Table 3). Abbreviations are listed in Table 4.

**Table 3:** Comparisons of the Corvis ST measurements between thin, normal, and thick corneas.

	Mean ± SD			*P*		
	Cornea_thin_	Cornea_normal_	Cornea_thick_	Cornea_thin_ VS Cornea_normal_	Cornea_thin_ VS Cornea_thick_	Cornea_normal_ VS Cornea_thick_
bIOP	14.72 ± 1.48	14.60 ± 1.55	14.72 ± 1.57	0.809	1.000	1.000
DA ratio 2	4.99 ± 0.42	4.51 ± 0.33	4.13 ± 0.29	< 0.001*	< 0.001*	< 0.001*
IR	9.27 ± 0.87	8.57 ± 0.91	7.55 ± 0.76	< 0.001*	< 0.001*	< 0.001*
ARTh	431.92 ± 77.08	456.55 ± 91.09	527.10 ± 92.06	0.140	< 0.001*	< 0.001*
SP-A1	79.21 ± 9.04	91.56 ± 9.79	102.54 ± 9.27	< 0.001*	< 0.001*	< 0.001*
Arc length	− 0.13 ± 0.02	− 0.14 ± 0.02	− 0.15 ± 0.02	0.004*	< 0.001*	0.038*
Defle A	1.00 ± 0.09	0.96 ± 0.09	0.90 ± 0.08	0.106	< 0.001*	< 0.001*
DefleA ratio 2	6.55 ± 0.77	5.73 ± 0.71	5.18 ± 0.72	< 0.001*	< 0.001*	0.001*
EMo	0.27 ± 0.07	0.26 ± 0.06	0.26 ± 0.06	0.329	0.437	0.965
ICR	0.16 ± 0.01	0.16 ± 0.02	0.14 ± 0.01	0.007*	< 0.001*	< 0.001*
A1L	2.19 ± 0.22	2.27 ± 0.22	2.45 ± 0.17	0.122	< 0.001*	< 0.001*
A1V	0.16 ± 0.02	0.15 ± 0.02	0.14 ± 0.02	0.009*	< 0.001*	0.002*
A1T	7.34 ± 0.24	7.53 ± 0.26	7.68 ± 0.28	< 0.001*	< 0.001*	0.002*
A2L	1.76 ± 0.19	1.89 ± 0.23	2.19 ± 0.29	0.001*	< 0.001*	< 0.001*
A2V	− 0.29 ± 0.02	− 0.29 ± 0.03	− 0.28 ± 0.02	0.464*	0.002*	0.059*
A2T	22.11 ± 0.30	22.11 ± 0.35	22.00 ± 0.34	1.000	0.141	0.151
HCT	17.28 ± 0.24	17.31 ± 0.26	17.30 ± 0.28	1.000	1.000	1.000
	5.13 ± 0.21	5.06 ± 0.24	4.94 ± 0.23	0.457	< 0.001*	0.002*
HCR	6.54 ± 0.54	6.88 ± 0.62	7.52 ± 0.54	0.002*	< 0.001*	< 0.001*
Def A	1.14 ± 0.10	1.11 ± 0.10	1.05 ± 0.08	0.229	< 0.001*	< 0.001*
CBI	0.55 ± 0.31	0.27 ± 0.26	0.05 ± 0.10	< 0.001*	< 0.001*	< 0.001*
TBI	0.52 ± 0.24	0.33 ± 0.26	0.23 ± 0.21	< 0.001*	< 0.001*	0.047*
SSI	0.87 ± 0.14	0.88 ± 0.13	0.96 ± 0.13	1.000	0.001*	0.001*

P values were adjusted for the age and SE difference. The adjusted age was 25.41 years, SE =  − 5.58D.

**Table 4:** A brief description of Corvis ST parameters.

bIOP	Biomechanically corrected IOP
CCT(c)	Central corneal thickness by Corvis ST
CCT(o)	Central corneal thickness by OCT
CCT(p)	Central corneal thickness by Pentacam HR
DA ratio	Maximum deformation amplitude ratio between the apex and at 2 mm from the apex
IR	Integrated radius
ARTh	Ambrósio’s Relational Thickness to the horizontal profile based on the thickness profile in the temporal-nasal direction. ARTh = corneal thickness thinnest/pachymetric progression
SP-A1	Stiffness parameter A1 uses the displacement between the apex in the undeformed state and the deflection at A1
Arc length	Highest concavity delta arc length
Defle A	Highest deflection amplitude
DefleA ratio	Deflection amplitude ratio between the apex and at 2 mm from the apex
EMo	Whole eyeball movement
ICR	Maximum inverse concave radius during the concave phase of the deformation response
A1L	First applanation length
A1V	First applanation velocity
A1T	Time to first applanation
A2L	Second applanation length
A2V	Second applanation velocity
A2T	Time to second applanation
HCT	Time to highest concavity
PD	Peak distance of highest concavity
HCR	Highest concavity radius
Def A	Highest deformation amplitude
CBI	Corneal biomechanical index (CBI) based on logistic regression analysis which combined deformation response parameters with corneal thickness profile. CBI = EXP (Beta)/(1 + EXP(Beta)) Where Beta = B1 × A1Velocity + B2 × ARTh + B3 × Stiffness parameter-A1 + B4 × DARatio1mm + B5 × DARatio2mm + B6 × SD–Deflection Amplitude + B7 and B1 = − 59.487, B2 = − 0.027; B3 = − 0.092, B4 = − 27.169, B5 = 5.472, B6 = − 0.599, B7 = 46.576
TBI	tomographic and biomechanical index (TBI), combined Scheimpflug-based corneal tomography and biomechanics for enhancing ectasia detection
SSI	stress strain index, based on the numerical modeling input and output parameters CCT, bIOP, and SP-HC(stiffness parameter at highest concavity)

## Discussion

Changes in corneal biomechanical properties are thought to appear prior to morphological changes in ectasia. Therefore, corneal biomechanical measurement is of significant importance in terms of early detection of corneal ectatic diseases such as keratoconus and post corneal refractive surgery ectasia. However, the measurement precision of new technology is of utmost importance. To the best of our knowledge, this is the first study, with a large sample size, to evaluate the repeatability of latest version of Corvis ST in healthy subjects with myopia seeking refractive surgery and stratifying by CCT. Our results showed that the repeatability for the majority of Corvis ST measurement parameters were good. The repeatability in the thick corneas appears to be marginally better than the normal and thin cornea groups. It is not clear why this difference was elicited in this study. The corneal deformation behavior, in particular, was significantly affected by corneal thickness. The agreement of CCT between Corvis ST and either Pentacam HR or OCT was poor with a mean difference of 20.16 μm (with LoA of 7.13 to 33.19) and 29.49 μm (with LoA of 15.26 to 43.72) which are too wide to be acceptable as clinically interchangeable. The Corvis ST and the Pentacam HR report the CCT at the corneal vertex normal and both devices use the same technique to determine this location. Despite this, the CCT measurement was not interchangeable between the two devices. The RTVue OCT reports the CCT as an average of the central 2 mm diameter of the scanned area. The device can be manually centred at any point, usually either the pupil centre or corneal vertex normal. In this study, it was manually centred on the corneal vertex normal. As this is different to the technique employed by the Corvis ST and Pentacam HR, this may be part of the reason for the discrepancy in the agreement between devices involving the RTVue.

The repeatability of CCT measurements with the Corvis ST was found to be excellent in previous studies^16–18^. Nemeth et al.^17^ showed an intraclass correlation coefficient (ICC) of CCT of 0.97 and Chen et al.^18^ reported S_w_ values of 3.57 μm and 8.16 μm for CCT in virgin and post laser-refractive corneas, respectively. In the current study, S_w_ and *r* for CCT measurements ranged 6.165 to 7.658 μm, and 17.078 to 21.213 μm respectively, in all groups. Similarly, IOP and bIOP also showed good repeatability in all three groups. The S_w_ and *r* for IOP and bIOP were < 1 mmHg (range 0.777 to 0.997 mmHg) and < 3 mmHg (range 2.153 to 2.761 mmHg) respectively. Lopes et al.^16^ found the S_w_ and *r* for IOP were 0.98 mmHg and 2.7146 mmHg, and the S_w_ and *r* for bIOP were 0.89 mmHg and 2.47 mmHg. Nemeth et al.^17^ found an ICC of 0.865 and coefficient of variation (CoV) of 6.9% for IOP. Chen et al.^18^ demonstrated repeatable measurements for IOP in both virgin and post-PRK eyes with S_w_ values of 0.59 and 0.55, and *r* values of 1.62 and 1.52, respectively. The bIOP algorithm was developed using numerous simulations with the Corvis-ST on human eye models with different CCT, age, topographies, material properties, and IOP values. This has been shown to have significantly reduced the reliance of IOP measurement on the above mentioned parameters^19^, and the bIOP correction has successfully provided close estimates of true IOP in ex-vivo tests conducted on human donor eye globes^20^. Also, Matsuura et al.^21^ found that the bIOP measurement from the Corvis ST is independent from CCT, akin to our study findings.

Dynamic corneal responses (DCRs) are defined as “deformation” parameters, while those from which whole eye movements are removed are described as “deflection” parameters. Some DCRs including Def A, DA ratio, defle A and defleA ratio at 2 mm showed good repeatability results in thick corneas. These findings were consistent with previous studies^16, 22^. Wu et al.^23^ found that the deformation amplitude (DA) exhibited excellent repeatability with a S_w_ of 0.098, which was similar to our result, albeit slightly higher. Nemeth et al.^17^ showed good repeatability for IOP and pachymetric values, whereas other measurements. Interestingly, our results demonstrated that a thinner CCT was associated with lower repeatability of measurements but perhaps without clinical significance, as shown by higher S_w_ and *r* values in general.

Researchers hypothesized that there was a decrease in the SP-A1, the displacement between the undeformed cornea and cornea at the first applanation, in keratoconus compared with normal eyes. They concluded that SP-A1 could be a potential marker when evaluating disease progression^24–26^. We found that the repeatability of SP-A1 in thin, normal and thick corneas were good. Ambrósio’s Relational Thickness to the horizontal profile (ARTh)^24, 27^ is based on the thickness profile in the temporal-nasal direction. We found that the S_w_ and *r* values of ARTh gradually reduced with increased CCT. The S_w_ values of ARTh were 59.239, 53.466 and 61.090 in thin, normal and thick cornea groups respectively, which appear poor.

The Corneal Biomechanical Index (CBI) is a parameter that aims to provide early detection of keratoconus, with a cut off value of 0.5^24^. In the current study, the S_w_ values for the CBI were 0.228, 0.157 and 0.076 in the thin, normal and thick corneas, respectively. The actual CBIs in this normal group of myopia subjects in the respective CCT groups were 0.55, 0.27 and 0.05. Hence, the thin cornea group in our study had a mean CBI value above the cut-off value for abnormality. The TBI is a combined parameter based on Scheimpflug-based corneal tomography and biomechanical assessments; generated by the leave-one-out cross-validation (RF/LOOCV) and is suggested to provide greater accuracy for detecting ectasia^28^. The TBI may be sensitive for detecting subclinical (fruste) ectasia among eyes with normal topography. In our study, the S_w_ values for TBI ranged from 0.070 to 0.094, demonstrating better repeatability than CBI values.

The stress strain index (SSI), is the corrected biomechanical index which has been shown to be independent of IOP and CCT^29^. We found no difference between values in the thin and normal corneal thickness groups. However, a significant difference between the thin or normal CCT and thick CCT was observed. The repeatability of SSI in three groups was very good, with S_w_ values of 0.056, 0.058 and 0.058 respectively.

In the evaluation of measurement repeatability, different examination intervals have been used among studies. Ali et al.^30^ compared Corvis ST measurements at the same time of a day across four different days, or at four times of a day, or taken 2 to 5 min apart. They found similar results for IOP, CCT and A1T with good repeatability. These results are comparable to the present study. However, in their study, DA, PD, A1V, A2V, A2T and HCT, had poorer repeatability than the present study. Ali et al. found A1L and A2L parameters had poor repeatability with all three intervals, which was similar to ours findings. Wang et al.^22^ assessed repeatability with 5-min intervals, and had an ICC of 0.98 for IOP, but they also found the A1L, A2L and HCT had poorer ICCs of 0.17, 0.21 and 0.43 which is in accordance with our findings. Interestingly, we observed better repeatability for PD and HCR in all three groups.

Previous studies have demonstrated that the majority of biomechanical metrics offered by the Corvis ST are significantly correlated to CCT, IOP, age, and SE^9, 11, 31–35^. However, the Corvis ST parameters are more affected by IOP than by CCT^9, 36^. We also demonstrated that the deformation parameters were negatively associated with CCT in normal eyes, which is a similar concept to the idea that a thicker cornea deforms less, and a thinner cornea deforms more^37, 38^. The main limitations of this study were the relatively small sample in one of the subgroups (thick cornea group) and the exclusion of patients with keratoconus and other corneal diseases. The latter will be a focus of future study.

In conclusion, overall the repeatability of the parameters measured with the Corvis ST were good with S_w_ values generally observed to be better in thicker corneas, with corneal deformation significantly affected by corneal thickness. Possible markers for the early detection of ectasia appear repeatable with the exception of CBI and ARTh. The parameter SSI appears promising parameter with differences observed between thin or normal corneal thickness and thick corneas. The CCT measurements with the Corvis ST were found not to be interchangeable with those acquired with the RTVue OCT and Pentacam HR.
